# Hospital to home transition of children with medical complexities in the Netherlands: current practice

**DOI:** 10.1007/s00431-024-05960-2

**Published:** 2025-01-08

**Authors:** H. N. Haspels, H. Knoester, N. J. G. Jansen, I. M. L. Ahout, C. D. van Karnebeek, M. de Hoog, J. B. M. vanWoensel, K. F. M. Joosten

**Affiliations:** 1 Department of Pediatric Intensive Care, Amsterdam UMC Location University of Amsterdam, Meibergdreef 9, Amsterdam, The Netherlands; 2 Amsterdam Reproduction & Development Research Institute, Amsterdam, The Netherlands; 3 Department of Neonatal & Pediatric Intensive Care, Division of Pediatric Intensive Care, Erasmus Medical Center – Sophia Children’s Hospital, Rotterdam, The Netherlands; 4 On Behalf of the Transitional Care Unit Consortium, Amsterdam, The Netherlands; 5 Department of Pediatrics, University Medical Center Groningen, Beatrix Children’s Hospital, Groningen, The Netherlands; 6 Department of Pediatrics, Radboud University Medical Center, Amalia Children’s Hospital, Nijmegen, The Netherlands; 7 Departments of Pediatrics and Human Genetics, Emma Center for Personalized Medicine, Amsterdam Reproduction and Development, Amsterdam UMC Location University of Amsterdam, Meibergdreef 9, Amsterdam, The Netherlands

**Keywords:** Hospital to home transition, Children with Medical Complexity, Care pathway, Transitional Care Unit

## Abstract

**Abstract:**

Children with Medical Complexity (CMC) often require 24/7 expert care, which may impede discharge from hospital to home (H2H) resulting in prolonged admission. Limited research exists on pediatric patients with delayed discharges and the underlying reasons for such extended admissions. Therefore, our objectives were to (1) describe the demographics, clinical characteristics, and course of CMC who are in their H2H transition and (2) identify the reasons for postponement of H2H discharge. Prospective, multicenter, observational cohort study performed from February 2022 until November 2022 for 6 months in four Dutch University Medical Center children’s hospitals. Clinically admitted patients (age 0–18 years) were eligible for inclusion if they were medically stable, yet required specialized nursing and/or paramedical care and were in the H2H transition process. In total, 44 participants were included, of whom 32 (72.7%) were younger than 1 year. Median stay in the hospital was 7.3 weeks (range 0.7–28.7). Upon entering the H2H phase, postponement of discharge was for 65.1% of the patients primarily due to a combination of medical reasons and organizational/family factors. For the remaining 34.9% of the patients, discharge was delayed solely due to organizational and/or family factors.

***Conclusion*:**

Our study highlights several reasons contributing to the postponement of discharge for pediatric patients with medical complexity, including their medical fragility, the time-consuming process of parent training, and the challenges in organizing home care. Future steps should explore various transitional care programs aimed at improving the H2H transition.

**Table:** 

**What is Known**:
• *Hospital to home transition for Children with Medical Complexity is a multi-faceted process with many challenges and obstacles*
• *Insight into the current practice of transitioning home in University Medical Centers remains unknown and is needed to develop a tailored yet standardized approach*
**What is New:**
• *Our findings reveal reasons for postponement of discharge home and show that patients are medically stable for more than half of their hospital stays. This indicates potential opportunities to reorganize care for better outcomes for the child, the family, and healthcare consumption*

**Supplementary Information:**

The online version contains supplementary material available at 10.1007/s00431-024-05960-2.

## Introduction

In the past decades, the survival rate of children with (critical) diseases has increased [1, 2]. This has resulted in a growing number of Children with Medical Complexity (CMC), whereby the focus is changed from *cure* to long-term *care* for child and family. Cohen et al. defined CMC as children having a chronic condition, high needs, functional limitations, and high healthcare use [1]. CMC often require 24/7 expert care resulting in prolonged (re)admissions in tertiary hospitals [3, 4]. These hospital admissions interfere with the psychosocial and motor development of the child and are disruptive to parental and family functioning and wellbeing [5]. In addition, prolonged hospital admissions are a burden for the hospital capacity and incur high costs [5, 6].

It is recognized that the transition from hospital to home (H2H) is a vulnerable period for CMC [7, 8]. The child’s primary caregivers, hereafter referred to as parents, evolve from care receivers in the hospital to the most important and responsible caregivers at home. Parents must be proficient in complex nursing care, deal with multiple healthcare providers, find a new balance in family and society, and need emotional support [9, 10]. Parents reported feeling unsupported and unprepared during the transition to their home environment [11–14].

Improving the organization of transitional care has become a prevalent issue, providing benefits to children, their families, and healthcare professionals [15]. Developing a tailored, yet standardized approach that improves H2H transition for CMC, their families, and healthcare professionals is crucial [16–18]. While interventions should be customized to meet the unique needs of each child and family (“tailored”), there are core components of transitional care that can be consistently applied across all cases (“standardized”). Put differently, many CMC families share common medical and non-medical needs, indicating that tailored interventions based on a uniform approach might benefit these families [9, 10]. However, scientific evidence for the best transitional care model is lacking or at best fragmented [19].

To improve care and create an optimized care pathway, it is essential to examine the current transitional care practices in relation to (parental) needs. So far, studies investigating prolonged hospital stays among CMC focus on specific groups, like ventilator-dependent children and those with tracheostomies, and found that non-medical factors, such as education and home nurse delays, largely impede discharge home [20, 21]. However, there is a significant knowledge gap regarding which pediatric patients experience delayed discharges and the underlying reasons for such extended admissions. These insights can give direction to potential beneficial interventions.

Therefore, the following research objectives were addressed: (1) to describe the demographics, clinical characteristics, and course of CMC who are in their H2H transition and experience a prolonged hospital stay and (2) to identify the reasons for postponement of H2H discharge.

## Methods

### Study design

Prospective, multicenter, observational cohort study performed in four Dutch UMCs children’s hospitals including the Amsterdam University Medical Center (Amsterdam UMC) – Emma Children’s Hospital, ErasmusMC (EMC) – Sophia Children’s Hospital, Radboudumc Nijmegen (Rumc) – Amalia Children’s Hospital, and University Medical Center Groningen (UMCG) – Beatrix Children’s Hospital. The study was carried out for 6 months in each center between February 2022 and November 2022.

The checklist of the STrengthening the Reporting of Observational studies in Epidemiology (STROBE) was used during the execution and reporting of the study [22].

### Study setting

In the Netherlands, most CMC have at least one pediatrician taking care of the child in one of the seven University Medical Centers (UMCs) (supplementary 1). In six of the seven UMCs, CMC typically transition directly from the hospital to home or a home-like setting. The exception is the Amsterdam UMC, where children can transition home through a Transitional Care Unit (TCU) called Jeroen Pit House (JPH) which is a stand-alone building on the premises of the hospital [23]. This TCU offers a unique approach, providing families with separate home-like apartments where they can practice caregiving under the direct available physically present supervision of healthcare professionals and adapt to their new reality before completing the transition home.

### Ethical considerations

Approval was provided by the Institutional Review Board of the Amsterdam UMC, location AMC, who waived the need for a full ethical review and concluded that, according to the national framework set by the Central Dutch Committee on Research in Humans (CCMO), the Medical Research Involving Human Subject Act does not apply to this study (reference number W21_581 # 22.025). The need for ethical approval was waived by all participating UMCs. Written informed consent was obtained from patients, patient’s parents, and/or legal guardians. All data were collected, analyzed, and reported anonymously, in compliance to the EU General Data Protection Regulation.

### Study participants

Clinically admitted patients (age 0–18 years) were screened once a week for eligibility throughout their stay in one of the participating UMCs. Patients were included if they met the following criteria: (1) older than 37 weeks corrected for gestational age and younger than 18 years of age, (2) admitted for more than 1 week in hospital or TCU, (3) stable medical condition or the treatment does not require daily adjustment, (4) future (expected) need of specialized medical care for at least 4 weeks, and (5) discharge home not yet possible.

The patient is defined as being in a stable medical condition if there is (1) a patent, sufficiently safe airway for the home situation, whether or not through a trachea cannula, (2) sufficient breathing, whether or not using (intermittent) support with oxygen, non-invasive ventilation, or invasive ventilation via a tracheal cannula, (3) a neurologically stable condition with temporary neurological impairments (such as seizures) not interfering (potentially life-threatening) with other vital functions such as respiration or circulation, and (4) drug treatment that can be given at home by (if applicable) a nasogastric tube, duodenal or jejunal tube, and/or a percutaneous endoscopic gastrostomy (PEG) tube, or a patented, “home-proof” intravenous access.

Patients were excluded if (1) they were in end-of-life care, (2) had predominantly social/family issues without serious medical problems, (3) had mainly behavioral/psychiatric problems, and (4) were in need of urgent rehabilitation medical care.

### Overall patient and family demographics

Demographics and pre-existing medical conditions, as documented in the medical record, were collected at enrollment. This included the following parameters: (gestational) age on admission, gender, diagnosis on admission, chronic complex condition (CCC), and technology support on admission. A CCC was defined according to the model of Feudtner et al. and Verlaat et al. [24, 25] (Supplementary 2). Additionally, the number of hospital (re)admissions, length of stay in the hospital, emergency visits, surgical procedures, and telephonic and outpatient consultations during the previous 12 months were collected in retrospect. Parents were asked to fill out a short questionnaire which included questions regarding age of the parents on admission, country of birth, ability to speak the Dutch language, parents living together, number of children in the family, and socio-economic status (educational and occupational level).

### Postponement of discharge

After enrollment, weekly reasons for postponement of transition home were recorded using a catalog (Supplementary 3). This was completed collaboratively with healthcare professionals (doctors and nurses) in the department. The H2H phase is defined in this article from the moment the patient achieves medical stability. Reasons for postponement of discharge were categorized into three groups: medical reasons, organizational factors (sustainable care plan, arrangement of home care, medical technology availability, safety of the home), and family factors (caregiver competencies, and emotional readiness).

Since patients are prone to relapse, the inclusion criteria for medical stability were weekly screened. Reasons for relapse were collected and patients were followed through hospital discharge.

### Discharge characteristics

The following parameters were collected upon discharge: discharge location, home care arrangements, medical day care or respite care, required technological support and medication after discharge, involved medical disciplines, cumulative length of stay in hospital and/or in TCU if applicable.

At discharge, patients were classified according to the model of Cohen concerning medical complexities [1]. Although abundant literature exists on CMC, a uniform classification system is lacking [26]. For this study, a child was classified as CMC when it met all four of Cohen’s domains: having a chronic condition, high family needs, functional limitations, and high healthcare use (Supplementary 1).

### Statistical analysis

All data was collected using Castor EDC [27]. Analyses were performed with the use of IBM SPSS Statistic, version 28. Variables were summarized as frequencies and percentages, median, and/or means.

## Results

### Participants

During the screening period, 91 patients were eligible, of whom 44 patients (48.4%) were included in the study. Reasons for exclusion were insurmountable language barriers (6.6%), unavailability for the informed consent procedure (17.6%), and parents not consenting (27.5%). The reasons for not consenting were that parents reported being already involved in multiple studies, and feeling overwhelmed. For the 44 included patients, baseline characteristics are reported in Table 1.

**Table 1:** Patient demographics

	Total *n* = 44 (%)
Female	23 (52.3)
Age in months	1.5 (0–200)
Age group	
< 1 month	19 (43.2)
1 month– < 1 year	13 (29.5)
1 year– < 10 years	8 (18.2)
10– < 18 years	4 (9.1)
Born premature	11 (25)
Admission type: elective	25 (56.8)
Admission diagnosis	
Surgical	
Gastrointestinal/abdominal	12 (27.9)
Cardiothoracic	2 (4.7)
Trauma/orthopedic	1 (2.3)
Medical	
Respiratory	9 (20.9)
Immunological/infectious	7 (16.3)
Gastrointestinal	11 (25.6)
Metabolic disorder	1 (2.3)
CCC	
None	4 (9.1)
One	22 (50)
Two	13 (29.5)
Three or more	5 (11.4)
Admitted from birth	25 (56.8)
Not admitted from birth but no prior medical history	3 (6.8)
Patients with medical history	16 (36.4)
Medical history*	
Technical assistance upon admission	14 (73.7)
Amount of technical assistance upon admission	1 (0–5)
Amount of (re)admissions**	2 (1–8)
Outpatients visits**	3 (0–21)
Emergency department visits**	1 (0–11)

Data are *n* (%) or median (range)

*CCC*, complex chronic condition

*From the 19 patients not admitted from birth

**Past 12 months including this admission

Median age was 1.5 months (range; 0–200), most patients were younger than 1 year of age (72.7%), and the majority of hospital admissions were elective (56.8%). The primary indications for admission were as follows: gastrointestinal/abdominal surgery (27.9%), gastrointestinal medical issues (25.6%), respiratory (20.9%) and infectious/immunological (16.3%) medical conditions. The patients often presented with a multitude of medical problems. Almost all patients (90.9%) had one or more (underlying) CCC. More elaborate information on their CCC and underlying medical conditions is presented in Supplementary 5 and 6.

The majority of patients (56.8%) were admitted to the hospital from birth onwards. Additionally, three patients (6.8%) had no prior medical history, while 16 (36.4%) did have a medical history. Among those with a medical history, most patients (73.7%) required technical assistance upon admission, with a median of one device (range 0–5). Furthermore, within the 12 months preceding admission, patients experienced (re)admissions (median 2; range 1–8), outpatient visits (median 3; range 0–21), and emergency department visits (median 1; range 0–11).

Regarding family characteristics, most patients (90%) lived with two parents, and the majority of parents spoke Dutch (93.4%) and were born in Western countries. Families had a median of two children (range; 1–5) (see Supplementary 4). Regarding socio-economic status, most participants had a middle educational level (55%), while the least represented group had a low educational level (17.5%). In terms of occupational level, the majority of participants were classified at Level 2 (30%) out of a total of 4 levels, with a significant proportion also at Level 1 (25%).

### The hospital to home phase

Forty-four patients entered the H2H; however, one patient (with a congenital diaphragmatic hernia) passed away unexpectedly. The remaining 43 patients had a median length of hospital stay of 7 weeks (range 0.7–26.7) (Table 2). Nine (out of the 10) patients from the Amsterdam UMC were referred to the TCU JPH. Patients referred to the TCU had a median hospital stay of 3.9 weeks (range 0.7–13.1) compared to patients not referred to the TCU, who had a median stay of 8.9 weeks (range 1.1–28.7). Patients had a median length of stay of 4.7 weeks (range 2–9) in the JPH.

**Table 2:** Hospital to home phase

	*N* (%)	Weeks, median (range)
Total length of hospital admission, weeks Length of hospital stay non TCU patients Length of hospital admission TCU patients Total length of stay in TCU, weeks	43 (100) 34 (79.1) 9 (20.9) 9 (20.9)	7.3 (0.7–28.7) 8.9 (1.1–28.7) 3.9 (0.7–13.1) 4.7 (2–9)
LOS in H2H phase in weeks	43 (100)	3 (1–25)
Properties H2H phase		
< 25% of total admission	7 (16.3)	
25–50% of total admission	13 (30.2)	
50–75% of total admission	16 (37.2)	
> 75% of total admission	7 (16.3)	
Unstable period	11 (25.6)	2 (1–6)
Reasons unstable period*	16 (100)	
Infections	8 (50)	
Stoma reversal	6 (14)	
Other type of surgery	2 (4.7)	

Data are *n* (%) or median (range)

*n*, number; *LOS*, length of stay; *H2H*, hospital to home

*One patient can have multiple reasons for experiencing an unstable period

The H2H phase lasted approximately weeks (range 1–25). Most patients (53.5%) were medically stable for more than half of the total length of their admission. During this H2H phase, 11 patients (25.6%) suffered from an unstable period, which lasted 2 weeks (median; range 1–6) and were mainly the result of infections (50%), stoma reversal (37.5%), or other type of surgery (12.5%).

### Postponement of discharge

Reasons for postponement of discharge are presented in Table 3. For most patients (65.1%), it was a combination of medical reasons and organizational/family factors. For the remaining patients, discharge was delayed solely due to organizational and/or family factors. Most medical reasons were related to optimizing (enteral) nutrition (41.9%), followed by intravenous antibiotic treatment (14%). Upon entering the H2H phase, a sustainable care plan had to be developed for 35 (81.4%) patients, home care arrangements for 33 (76.6%) patients, and medical technology arrangements for 32 (74.4%) patients. This was arranged within a median (range) of 3 (1–16) weeks after entering the H2H phase. In 7 (16.3%) households, their home was not safely equipped for their sick child and it took a median (range) of 4 (2–17) weeks before this was solved. Almost all parents (81.4%) needed to learn how to take care of their child, which took a median of 3 weeks (range 1–24). Lastly, in 4 families (9.3%), everything was arranged and parents were trained; however, they were emotionally not ready to go home, resulting in a prolonged stay (median (range) 1 week (1–1)).

**Table 3:** Reasons for postponement of discharge

		*N* (%)	Weeks, median (range)
Medical reasons	Medical reasons*	28 (65.1)	2.5 (1–16)
	Specific reasons**		
	Intravenous antibiotic treatment	6 (14)	
	Optimizing (enteral) nutrition	18 (41.9)	
	High flow nasal cannula weaning	5 (11.6)	
	Reduce suctioning	1 (2.3)	
	High output stoma	2 (4.7)	
	Monitoring oxygen saturation	1 (2.3)	
Organizational factors	Sustainable care plan	35 (81.4)	3 (1–25)
	Arrangement of home care	33 (76.7)	3 (1–25)
	Medical technology availability	32 (74.4)	3 (1–25)
	Safety of the home	7 (16.3)	4 (2–17)
Family factors	Caregiver competencies	35 (81.4)	3 (1–24)
	Emotional readiness	4 (9.3)	1 (1–1)

Data are *n* (%) or median (range)

*n*, number; *LOS*, length of stay; *H2H*, hospital to home

*Medical reasons in combination with organizational/family

**One patient can have multiple medical reasons for postponement of discharge

### Care at home

Of the 43 patients, 36 (83.7%) were discharged directly home (Table 4). Two patients were transferred to a medical care facility outside the hospital before transiting home. Four of the remaining five children were transferred to another (non) academic hospital, while one patient was discharged to the Ronald McDonald House. In total, 33 children (76.7%) could be classified as CMC. Specifically, upon discharge, 83.7% of all patients were equipped with one or more medical devices, and were prescribed a median of 5 (range 0–28) medications for use at home. Engagement with healthcare professionals post-discharge was ensured for all patients, with a median of 3 medical specialists (range 0–6) and a median of 2 allied healthcare professionals (range 0–4) involved after discharge. Once home, 27 patients (62.8%) received home nursing care, 5 patients (11.6%) attended a medical daycare facility (with 4 of these also receiving home care), and 4 patients (9.3%) received respite care in addition to home care.

**Table 4:** Discharge characteristics

	Total *n* = 43 (%)
Discharge location	
Home	36 (83.7)
Medical care facility	2 (4.7)
Other (non)academic hospital	4 (9.3)
Ronald McDonald House	1 (2.3)
CMC	33 (76.7)
Functional limitations	
Technical devices	36 (83.7)
Monitor	9 (20.9)
Tracheostomy	4 (9.3)
Mechanical ventilation	4 (9.3)
Oxygen therapy	4 (9.3)
Coughing device	3 (7)
Central line	8 (18.6)
Stoma	2 (4.7)
Gastrostromy/jejunostomy	5 (11.6)
Nasogastric tube	21 (48.8)
Nasopharyngeal tube	1 (2.3)
Hearing aid	1 (2.3)
Amount of medication at home	5 (0–28)
Involvement HCP after discharge	43 (100)
Medical specialists	3 (0–6)
Allied healthcare professionals	2 (0–4)
Home care	27 (62.8)
Medical daycare	5 (11.6)
Respite care	4 (9.3)

Data are *n* (%) or median (range)

*n*, number; *CMC*, Children with Medical Complexity; *HCP*, healthcare professionals

## Discussion

Our prospective study focused on the transition for pediatric patients admitted for more than 1 week in the Netherlands from four University Medical Center Children’s Hospitals to home. These patients were medically stable but still required nursing and/or paramedical care and were not yet ready for discharge home. Our findings reveal that this group faces a wide range of (complex) medical challenges. Delays in discharge were due to both medical and organizational or family factors, with most patients spending over half of their hospital stay in a medically stable condition. When finally discharged, approximately three quarter of the patients could be classified as CMC requiring (complex) chronic home care.

### The hospital to home phase

Patients in this study were admitted to the hospital for a long period which is in line with other studies [4, 28]. In our study, most patients were medically stable more than half of the total length of stay in the hospital, implying that earlier discharge could be possible. This is concerning since longer hospitalization has been associated with negative parental outcomes such as stress, post-traumatic disorder, depression, anxiety [29–32], and a disruption of parents-child bonding [33]. Furthermore, it can be particularly frustrating for parents when their child is medically stable, as they are eager to go home [8]. Price et al. revealed that once technology-dependent children achieved medical stability, the hospital setting was not ideal as their family lives were “on hold.” Transitioning to a home-like step-down care facility allowed both parent(s) and child to “live again” instead of merely “exist” [34].

Several studies on interventions aimed at reducing hospitalizations for CMC focus on expanding access to familiar providers, enhancing caregiver knowledge and skills, developing proactive crisis or contingency plans, and improving transitions between hospital and home [35]. Additionally, post-acute care facilities could help reduce the length of hospital stays, particularly for stable children who still require ongoing care and supervision from nurses or other healthcare professionals. This is supported by studies showing that children with tracheostomies spend fewer days in acute care hospitals when post-acute care facilities are utilized [36, 37]. Beyond the scope of our study, we observed that patients transitioned to the TCU JPH had shorter hospital stays compared to those going directly home, averaging 8.9 days versus 3.9 days, respectively. However, due to the limited sample size, we cannot draw definitive conclusions about the significance of these differences, highlighting the need for further research.

### Postponement of discharge

In our study, non-medical reasons for discharge postponement included the time and effort required for parents to acquire specific (technical) skills needed for their child’s care. This finding aligns with other studies, which suggest that due to the extensive range of material to be covered, it is crucial to start training as early as possible during a child’s hospital stay [38, 39]. However, this contrasts with the challenges of training parents who may not always be present at the hospital due to commitments such as caring for other children or work obligations [40]. Therefore, it is essential to collaborate with parents in developing a feasible educational plan and a setting that enables them to effectively integrate various activities, thereby minimizing prolonged hospital stays.

Additionally, some families were unable to return home because the necessary care at home was not yet properly organized. The challenges of coordinating care across the hospital setting are frequently discussed, highlighting the need for improvements that benefit both parents and healthcare professionals [41, 42]. This process can be time-consuming and complex, as hospital healthcare professionals are often unfamiliar with available home (health) care services or how to arrange them [43]. In addition, once the patient is home, different organizations are responsible for the care often leading to communication barriers between the hospital-based and home care services [44]. Various approaches, such as utilizing an advanced nurse practitioner, are being explored to improve care coordination [45]. A standardizing discharge plan could assist in identifying available care options [46]. In specific populations, such as ventilator-dependent children, standardized discharge care has already led in reduced length of stay, fewer readmissions, and improved caregiver satisfaction [47]. In the Netherlands, there is currently no established care pathway for transition care in CMC. Given these findings, exploring the development of such a pathway would be advantageous.

Lastly, several families in our study experienced prolonged hospital stays due to their homes not being adequately equipped to meet their child’s needs. Inadequate housing, such as the lack of accessible facilities or navigating stairs with a disabled child, is a common issue [48]. Smith et al. emphasize that discussions about housing needs between families and healthcare providers are often limited, although such conversations can offer valuable insights [49]. Addressing these challenges enables healthcare providers to tailor care plans and referrals, ensuring transitional care is aligned with the family’s specific needs.

### Family characteristics

In our study, we observed an underrepresentation of one-parent households, non-Western parents, non-Dutch speakers, and individuals with lower education levels. Currently, there is no existing literature on the impact of social determinants of health on H2H transitions for CMC. However, language barriers, for example, can lead to poorer access to healthcare, longer lengths of stay, higher costs, and increased rates of readmission for CMC [50, 51]. Financial and social hardships might significantly affect H2H transitions. For example, when a parent in a single-parent household becomes ill or must care for other siblings, this can reduce time available for training and lead to extended hospitalizations. Future studies should explore these social determinants of health and their influence on H2H transitions for CMC, as understanding these factors is crucial for improving care and support for families.

### Strengths and limitations

The strength of this study is that it is a prospective multicenter study, encompassing more than 65% of pediatric UMC hospital beds in the Netherlands. This is the first study to identify reasons for postponement in the H2H transition process of CMC in the Netherlands, identifying potential areas for improvement. A limitation of this study is the small sample size, which was expected given the low number of CMC in the Netherlands. However, we also observed a high rate of non-participation. This may result in an incomplete population representation which does not fully capture the diversity of the population. A final limitation is that, for practical reasons, we conducted weekly screenings and scored the reasons for discharge postponement. If the study had been extended over a longer period with daily assessments, it likely would have yielded a more comprehensive and detailed understanding of the situation.

## Conclusion

Our study highlights several reasons contributing to the postponement of discharge for pediatric patients with medical complexity, including their medical fragility, the time-consuming process of parent training, and the challenges in organizing home care. Future research should explore various transitional care programs aimed at improving the H2H transition, such as TCUs or standardized discharge processes. Another relevant research avenue might well be applying a healthcare modularity perspective [52] for improving the organization of transitional care since “tailored yet standardized” is essential.

## Supplementary Information

Below is the link to the electronic supplementary material.

Supplementary file1 (DOCX 37 KB)

## Data Availability

No datasets were generated or analyzed during the current study.

## Abbreviations

CCC: Chronic complex condition
CMC: Children with Medical Complexity
H2H: Hospital to home
JPH: Jeroen Pit Huis
STROBE: STrengthening the Reporting of Observational studies in Epidemiology
TCU: Transitional Care Unit
UMCs: University Medical Centers

## References

[1] Cohen E et al (2011) Children with medical complexity: an emerging population for clinical and research initiatives. Pediatrics 127(3):529–538 21339266 10.1542/peds.2010-0910 PMC3387912

[2] Fraser LK et al (2012) Rising national prevalence of life-limiting conditions in children in England. Pediatrics 129(4):e923–e929 22412035 10.1542/peds.2011-2846

[3] Cohen E et al (2012) Patterns and costs of health care use of children with medical complexity. Pediatrics 130(6):e1463–e1470 23184117 10.1542/peds.2012-0175 PMC4528341

[4] Gold JM et al (2016) Long length of hospital stay in children with medical complexity. J Hosp Med 11(11):750–756 27378587 10.1002/jhm.2633

[5] Nelson LP, Gold JI (2012) Posttraumatic stress disorder in children and their parents following admission to the pediatric intensive care unit: a review. Pediatr Crit Care Med 13(3):338–347 21499173 10.1097/PCC.0b013e3182196a8f

[6] Kokorelias KM et al (2019) Towards a universal model of family centered care: a scoping review. BMC Health Serv Res 19(1):1–11 31409347 10.1186/s12913-019-4394-5 PMC6693264

[7] Leyenaar JAK et al (2017) Families’ priorities regarding hospital-to-home transitions for children with medical complexity. Pediatrics 139(1):e20161581 27940509 10.1542/peds.2016-1581 PMC5192089

[8] Curran JA, Breneol S, Vine J (2020) Improving transitions in care for children with complex and medically fragile needs: a mixed methods study. BMC Pediatr 20:1–14 32410674 10.1186/s12887-020-02117-6 PMC7222504

[9] van de Riet L et al (2023) Delineating family needs in the transition from hospital to home for children with medical complexity: part 1, a meta-aggregation of qualitative studies. Orphanet J Rare Dis 18(1):386 38082309 10.1186/s13023-023-02942-9 PMC10714518

[10] van de Riet L., et al (2023) Delineating family needs in the transition from hospital to home for children with medical complexity: part 2, a phenomenological study. Orphanet J Rare Dis 18:387. 10.1186/s13023-023-02747-w 10.1186/s13023-023-02747-w PMC10714565 38082332

[11] Nicholl HM, Begley CM (2012) Explicating caregiving by mothers of children with complex needs in Ireland: a phenomenological study. J Pediatr Nurs 27(6):642–651 23101728 10.1016/j.pedn.2011.07.003

[12] Frush JM., Ming DY, Crego N, Paden ME, Jones-Hepler B, Misiewicz R, Jarrett VA, Docherty SL (2023) Caregiver perspectives on telemedicine for postdischarge care for children with medical complexity: a qualitative study. J Pediatric Health Care 37(4):356–363. 10.1016/j.pedhc.2022.12.009 10.1016/j.pedhc.2022.12.009 PMC10330386 36670018

[13] Desai AD et al (2016) Caregiver perceptions of hospital to home transitions according to medical complexity: a qualitative study. Acad Pediatr 16(2):136–144 26703883 10.1016/j.acap.2015.08.003

[14] Zanello E et al (2015) Continuity of care in children with special healthcare needs: a qualitative study of family’s perspectives. Ital J Pediatr 41(1):1–9 25882884 10.1186/s13052-015-0114-x PMC4328636

[15] Pordes E et al (2018) (2018) Models of care delivery for children with medical complexity. Pediatrics 141(Supplement_3):S212–S223 29496972 10.1542/peds.2017-1284F

[16] Nageswaran S, Sebesta MR, Golden SL (2020) Transitioning children with medical complexity from hospital to home health care: implications for hospital-based clinicians. Hosp Pediatr 10(8):657–662 32631842 10.1542/hpeds.2020-0068

[17] Brenner M et al (2020) Key constituents for integration of care for children assisted with long-term home ventilation: a European study. BMC Pediatr 20(1):71 32061253 10.1186/s12887-020-1979-4 PMC7023713

[18] Brenner M et al (2018) Principles for provision of integrated complex care for children across the acute-community interface in Europe. Lancet Child Adolescent Health 2(11):832–838 30336897 10.1016/S2352-4642(18)30270-0

[19] Breneol S et al (2017) Strategies to support transitions from hospital to home for children with medical complexity: a scoping review. Int J Nurs Stud 72:91–104 28521207 10.1016/j.ijnurstu.2017.04.011

[20] Graf JM et al (2008) Children with new tracheostomies: planning for family education and common impediments to discharge. Pediatr Pulmonol 43(8):788–794 18613098 10.1002/ppul.20867

[21] Sobotka SA et al (2019) Attributable delay of discharge for children with long-term mechanical ventilation. J Pediatr 212:166–171 31153586 10.1016/j.jpeds.2019.04.034 PMC7290238

[22] Vandenbroucke JP et al (2007) Strengthening the Reporting of Observational Studies in Epidemiology (STROBE): explanation and elaboration. Annal Intl Med 147(8):W-163-W−194 10.7326/0003-4819-147-8-200710160-00010-w1 17938389

[23] Het Jeroen Pit Huis (2022) https://hetjeroenpithuis.nl/. Accessed 10/12/2024

[24] Verlaat CW et al (2017) Factors associated with mortality in low-risk pediatric critical care patients in the Netherlands. Pediatr Crit Care Med 18(4):e155–e161 28178075 10.1097/PCC.0000000000001086

[25] Feudtner C et al (2014) Pediatric complex chronic conditions classification system version 2: updated for ICD-10 and complex medical technology dependence and transplantation. BMC Pediatr 14(1):1–7 25102958 10.1186/1471-2431-14-199 PMC4134331

[26] Berry JG et al (2015) Ways to identify children with medical complexity and the importance of why. J Pediatr 167(2):229–237 26028285 10.1016/j.jpeds.2015.04.068 PMC5164919

[27] Castor E Castor electronic data capture. Ciwit BV, Amsterdam. https://castoredc.com. Accessed 10/12/24

[28] Boerman GH et al (2023) Characteristics of long-stay patients in a PICU and healthcare resource utilization after discharge. Critical Care Explorations 5(9):e0971 37644970 10.1097/CCE.0000000000000971 PMC10461958

[29] Diaz-Caneja A et al (2005) A child’s admission to hospital: a qualitative study examining the experiences of parents. Intensive Care Med 31(9):1248–1254 16021417 10.1007/s00134-005-2728-8

[30] Knoester H, Grootenhuis MA, Bos AP (2007) Outcome of paediatric intensive care survivors. Eur J Pediatr 166(11):1119–1128 17823815 10.1007/s00431-007-0573-1 PMC2039787

[31] Fink EL et al (2020) A core outcome set for pediatric critical care. Crit Care Med 48(12):1819–1828 33048905 10.1097/CCM.0000000000004660 PMC7785252

[32] Fauman KR et al (2011) Predictors of depressive symptoms in parents of chronically ill children admitted to the pediatric intensive care unit. Am J Hospice Palliative Med 28(8):556–563 10.1177/1049909111403465 21454321

[33] Noyes J (2000) ‘Ventilator-dependent’ children who spend prolonged periods of time in intensive care units when they no longer have a medical need or want to be there. J Clin Nurs 9(5):774–783

[34] Price J, McCloskey S, Brazil K (2018) The role of hospice in the transition from hospital to home for technology-dependent children—a qualitative study. J Clin Nurs 27(1–2):396–406 28658513 10.1111/jocn.13941

[35] Coller RJ et al (2017) Strategies to reduce hospitalizations of children with medical complexity through complex care: expert perspectives. Acad Pediatr 17(4):381–388 28108374 10.1016/j.acap.2017.01.006

[36] Feudtner C et al (2005) Technology-dependency among patients discharged from a children’s hospital: a retrospective cohort study. BMC Pediatr 5(1):1–8 15882452 10.1186/1471-2431-5-8 PMC1142327

[37] Berry JG et al (2009) Predictors of clinical outcomes and hospital resource use of children after tracheotomy. Pediatrics 124(2):563–572 19596736 10.1542/peds.2008-3491 PMC3614342

[38] Sterni LM et al (2016) An official American Thoracic Society clinical practice guideline: pediatric chronic home invasive ventilation. Am J Respir Crit Care Med 193(8):e16–e35 27082538 10.1164/rccm.201602-0276ST PMC5439679

[39] Sobotka SA, Hird-McCorry LP, Goodman DM (2016) Identification of fail points for discharging pediatric patients with new tracheostomy and ventilator. Hosp Pediatr 6(9):552–557 27538680 10.1542/hpeds.2015-0277

[40] Deavin A, Greasley P, and Dixon C (2018) Children’s perspectives on living with a sibling with a chronic illness. Pediatrics 142(2):e20174151. 10.1542/peds.2017-4151 10.1542/peds.2017-4151 30049891

[41] Foster CC et al (2017) Provider perspectives of high-quality pediatric hospital-to-home transitions for children and youth with chronic disease. Hosp Pediatr 7(11):649–659 29038132 10.1542/hpeds.2017-0031

[42] Ronan S, Brown M, Marsh L (2020) Parents’ experiences of transition from hospital to home of a child with complex health needs: a systematic literature review. J Clin Nurs 29(17–18):3222–3235 32621293 10.1111/jocn.15396

[43] Carter B et al (2016) “Knowing the places of care”: how nurses facilitate transition of children with complex health care needs from hospital to home. Comprehen Child Adolescent Nurs 39(2):139–153

[44] Altman L et al (2018) A qualitative study of health care providers’ perceptions and experiences of working together to care for children with medical complexity (CMC). BMC Health Serv Res 18(1):1–11 29386026 10.1186/s12913-018-2857-8 PMC5793356

[45] Looman WS et al (2013) Care coordination for children with complex special health care needs: The value of the advanced practice nurse’s enhanced scope of knowledge and practice. J Pediatr Health Care 27(4):293–303 22560803 10.1016/j.pedhc.2012.03.002 PMC3433641

[46] Statile AM, Schondelmeyer AC, Thomson JE, Brower LH, Davis B, Redel J, Hausfeld J, Tucker K, White DL, White CM (2016) Improving discharge efficiency in medically complex pediatric patients. Pediatrics 138(2):e20153832. 10.1542/peds.2015-3832 10.1542/peds.2015-3832 27412640

[47] Baker CD, Martin S, Thrasher J, Moore HM, Baker J, Abman SH, Gien J (2016) A standardized discharge process decreases length of stay for ventilator-dependent children. Pediatrics 137(4):e20150637. 10.1542/peds.2015-0637 10.1542/peds.2015-0637 PMC4811306 26966133

[48] Esser K et al (2022) Housing need among children with medical complexity: a cross-sectional descriptive study of three populations. Acad Pediatr 22(6):900–909 34607051 10.1016/j.acap.2021.09.018

[49] Smith BM, Donohue PK, Seltzer RR (2024) Family perspectives on provider conversations about housing needs for children with medical complexity. Child Care Health Dev 50(2):e13253 38529766 10.1111/cch.13253

[50] Eneriz-Wiemer M et al (2014) Parental limited English proficiency and health outcomes for children with special health care needs: a systematic review. Acad Pediatr 14(2):128–136 24602575 10.1016/j.acap.2013.10.003

[51] Thomson J et al (2016) Financial and social hardships in families of children with medical complexity. J Pedia 172:187–193 10.1016/j.jpeds.2016.01.049 PMC4846519 26897040

[52] Lennips A et al (2024) Continuity of care for children with anorexia nervosa in the Netherlands: a modular perspective. Eur J Pediatr 183(5):2463–2476 38470519 10.1007/s00431-024-05497-4 PMC11035398

