# Supplementary material for: Hospital to home transition of children with medical complexities in the Netherlands: current practice
Source: Eur J Pediatr. 2025 Jan 8;184(1):122. doi: 10.1007/s00431-024-05960-2 (PMC11711838; doi:10.1007/s00431-024-05960-2)
Supplement: Supplementary file 1 — Supplementary file1 (DOCX 37 KB) [file 431_2024_5960_MOESM1_ESM.docx]

**Supplementary 1: Additional information University Medical Centers (UMCs) in the Netherlands**

In the Netherlands, seven University Medical Centers (UMCs) provide specialized care for Children with Medical Complexity (CMC). Among these, the Amsterdam UMC's Emma Children's Hospital is the largest, with 164 beds, followed by the Sophia Children's Hospital at Erasmus MC in Rotterdam with 160 beds. The Wilhelmina Children's Hospital at the University Medical Center Utrecht has 134 beds, while the Radboud University Medical Center’s Amalia Children’s Hospital in Nijmegen has 101 beds. The Beatrix Children’s Hospital at the University Medical Center Groningen has 92 beds, the MoSa Kids Children’s Hospital at Maastricht University Medical Center has 65 beds, and lastly, the Willem Alexander Children’s Hospital in Leiden has 50 beds.

All of these hospitals are equipped with a pediatric intensive care unit (ICU), and various general pediatric departments, ensuring comprehensive care for both critically ill and less acute pediatric patients.

**Supplementary 2: Complex Chronic Conditions (CCC) and Children with Medical Complexity (CMC)**

*Complex Chronic Condition (CCC)*

A complex chronic condition (CCC) was defined according to Feudtner et al. [1] as “any medical condition that can be reasonably expected to last at least 12 months (unless death intervenes) and to involve either several different organ systems or one organ system severely enough to require specialty pediatric care and probably some period of hospitalization in a tertiary care center.”

A CCC classification was assigned when the primary admission diagnosis, underlying diagnosis, and/or comorbidities aligned with diagnoses categorized as CCCs based on a modified list developed by Feudtner et al. and Verlaat et al., [2]. Additionally, we quantified the number of CCCs for each pediatric patient and grouped them into categories ranging from 1 to 3 or more.

*Children with Medical Complexity (CMC)*

Cohen et al. (2011) defined CMC as children having a chronic condition, high needs, functional limitations and high healthcare use [3]. In this study, a child was classified as CMC when it met all four domains:

1. The chronic condition domain was met if the child was diagnosed with a complex chronic condition according to the model of Feudtner and Verlaat et al., and/or if there was (an expected) continuous dependence on technology for at least six months after discharge [1, 4]. An unknown but suspected complex and chronic condition, such as a child born with multiple congenital anomalies but lacking a unifying diagnosis, will also be considered to meet the criteria for this domain.
2. The functional limitation domain was met if patients were discharged with one of the following technological devices; a tracheostomy, mechanical ventilation, oxygen therapy, airway clearance intervention techniques, monitoring of oxygen saturation, IV therapy medications (total parenteral nutrition, medication through central or PICC line), feeding tube (nasogastric tube, gastrostomy and jejunostomy), colostomy and wheelchair.
3. The domain of high family needs was met if patients were discharged with three or more different types of medications and/or if two or more allied health care professionals were kept involved in primary or outpatient care (dietician, physio-, occupational- and, speech- therapist).
4. The domain for high health care use was met if patients had a stay in the hospital >14 days in the past year

**Supplementary 3: List of reasons for postponement of discharge**

| Category | Sub category | Question | Yes | No |
| --- | --- | --- | --- | --- |
| Medical reasons |  | Are there any medical reasons why the child cannot go home yet?  If yes, please specify: |  |  |
| Organizational factors | Sustainable care plan | Has a sustainable care plan been drawn up that includes at least: (I) care needs (II) clear coordination of tasks and responsibilities of parents and healthcare professionals? |  |  |
|  | Arrangement of home care | Is home care support arranged, or will it be arranged in the short term (within 2 weeks after discharge)? |  |  |
|  | Medical technology availability | Is essential medical technology available and deployable for care at home? |  |  |
|  | Safety of the home | Is the house located and equipped in a way that ensures the child's safety (accessibility for emergency services, parents' phone accessibility, technical facilities) |  |  |
| Family factors | Caregiver competencies | Have the parents/caregivers been certified in care competencies? Specifically, in terms of their ability to:  (i) assess their child for somatic deteriorations,  (ii) respond in emergency situations (e.g., seeking assistance, performing resuscitation, and, if applicable, dealing with issues like dislocated tracheostomy tubes, PEG tube problems, or seizure management), and  (iii) determine when to seek help and know who to call. |  |  |
|  | Emotionally readiness | Are parents/caregivers, in consultation with healthcare professionals, mentally and emotionally prepared to handle a new home situation? |  |  |

**Supplementary 4: Family characteristics**

Of the 44 children, parents of 40 children filled in the questionnaire about family demographics. In total, 76 parents are caring for 40 children.

| **Two-parent families living together**  **Single-parent families** | 36 (90)  4 (10) |
| --- | --- |
| **Age in years*** | 34 (21-49) |
| **Dutch speaking*** | 71 (93.4) |
| **Country of birth***  Netherlands  Other European Nations  African Nations  South Asian Nations  Caribbean | 59 (77.6)  5 (6.6)  6 (7.9)  3 (3.9)  3 (3.9) |
| **Socio-economic status**  Educational level  Level 1  Level 2  Level 3  Missing  Occupational level  Level 1  Level 2  Level 3  Level 4  Missing | 7 (17.5)  22 (55)  10 (25)  1 (2.5)  10 (25)  12 (30)  8 (20)  4 (10)  6 (15) |
| **Amount of children in the family** | 2 (1-5) |
| **Ranking index child in the family**  Only child  Youngest of the two children  Youngest of the three children  Second of the three children  Third of the four children  Oldest of the five children  Youngest of the five children | 16 (40)  15 (37.5)  4 (10)  1 (2.5)  2 (5)  1 (2.5)  1 (2.5) |
| Data are n (%) or median (range)  * Based on 76 parents  * Educational level of all interviewed parents based on CBS data (statline.cbs.nl): low (1), middle (2), and high (3) educational level. The average score of the paternal and maternal educational level was calculated and categorized as: average 1 or 1.5 = category 1, average 2 or 2.5 = category 2, average 3 = category 3.  ** Occupational level of all interviewed parents categorized according to the ISCO system for professions ([http://www.ilo.org/public/english/bureau/stat/isco](about:blank)): The average score of the paternal and maternal occupational level was calculated and categorized as: average 1 or 1.5 = category 1, average 2 or 2.5 = category 2, average 3 or 3.5 = category 3, average 4 = category 4. | |

**Supplementary 5: More specific overview of the Complex Chronic Conditions (CCC)**

The 44 patients had a total of 63 CCC classified according to the Feudtner and Verlaat model [1, 2]. Table 1 lists the specific conditions.

Table 1 Specific Complex Chronic Conditions

|  | **(n=63)** |
| --- | --- |
| Neurologic and neuromuscular  CNS degeneration and disease  Brain and spinal cord malformations  Epilepsy  Other disorders of CNS | 2  2  2  1 |
| Cardiovascular  Heart and great vessel malformations  Endocardium disease | 9  1 |
| Respiratory  Respiratory malformations  Cystic Fibrosis  Chronic respiratory diseases  Devices | 3  3  2  2 |
| Gastrointestinal  Congenital anomalies | 6 |
| Hematologic or immunologic  Leukopenia  Hereditary anemias | 1  1 |
| Metabolic / Endocrine  Lipid metabolism  Endocrine disorders | 1  1 |
| Other congenital or genetic defects  Bone and joint anomalies  Diaphragm and abdominal wall  Chromosomal anomalies  Other anomalies | 6  10  3  2 |
| Premature and neonatal  Birth asphyxia  Other | 1  4 |
| Data are n  CNS, central nervous system | |

| **Supplementary 6: Primary underlying medical condition** | |
| --- | --- |
|  | **Demographics (n=44)** |
| Neurologic and neuromuscular  Neurodegenerative disorder (e causa Ignota)  Dandy-Walker Syndrome  Schaaf-Yang Syndrome  Rett syndrome  Meningitis  Post anoxic encephalopathy | 1  1  1  1  1  1 |
| Cardiovascular  Pulmonary artery sling  Congenital heart disease | 1  1 |
| Respiratory  Cystic fibrosis  Tracheomalacia | 3  1 |
| Gastrointestinal  M. Hirschsprung  Gastroschisis  Esophageal atresia  Ileum atresia  Congenital Hernia Diaphragmatica  Omphalocele  Necrotizing enterocolitis  Meconium plug  Milk curd syndrome  Spontaneous Intestinal Perforation | 2  3  1  1  6  1  2  1  1  1 |
| Immunologic / infectious  HIV infection  Late onset neonatal sepsis  Intracranial infection | 1  1  1 |
| Metabolic / Endocrinologic  CDG syndrome | 1 |
| Other genetic defects  Stickler syndrome  22q11 syndrome  Kabuki syndrome  CHARGE  Trisomy 21  Multiple congenital abnormalities (e cause Ignota) | 1  1  1  1  1  3 |
| Others  Trauma, orthopedic | 1 |
| Data are n. IV; Human Immunodeficiency Virus, CDG; Congenital Disorder of Glycosylation, CHARGE; Coloboma, Heart defects, Atresia of the choanae, Retardation of growth and development, Genital abnormalities, Ear abnormalities | |

**References supplementary**

1. Feudtner, C., et al., *Pediatric complex chronic conditions classification system version 2: updated for ICD-10 and complex medical technology dependence and transplantation.* BMC pediatrics, 2014. **14**(1): p. 1-7.

2. Verlaat, C.W., et al., *Factors associated with mortality in low-risk pediatric critical care patients in the Netherlands.* Pediatric critical care medicine, 2017. **18**(4): p. e155-e161.

3. Cohen, E., et al., *Children with medical complexity: an emerging population for clinical and research initiatives.* Pediatrics, 2011. **127**(3): p. 529-538.

4. Simon, T.D., et al., *Pediatric medical complexity algorithm: a new method to stratify children by medical complexity.* Pediatrics, 2014. **133**(6): p. e1647-e1654.
